# MicroRNA‐191 promotes hepatocellular carcinoma cell proliferation by has_circ_0000204/miR‐191/KLF6 axis

**DOI:** 10.1111/cpr.12635

**Published:** 2019-07-23

**Authors:** Fang Tian, Chengtao Yu, Min Wu, Xiaoyu Wu, Lingfeng Wan, Xuejun Zhu

**Affiliations:** ^1^ Affiliated Hospital of Nanjing University of Chinese Medicine, Jiangsu Province Hospital of Chinese Medicine Nanjing China; ^2^ School of Biomedical Engineering Shanghai Jiao Tong University Shanghai China; ^3^ Life Science and Technology Institute, China Pharmaceutical University Nanjing China

**Keywords:** cell cycle, has_circ_0000204, Hepatocellular carcinoma, KLF6, miR‐191

## Abstract

**Objectives:**

MicroRNAs are powerful regulators in hepatocellular carcinoma (HCC) tumorigenesis. MicoRNA‐191 (miR‐191) has been reported to play an important role in HCC, However, the regulatory mechanism is still unclear. In this study, we investigated the role of miR‐191 in HCC and studied its underlying mechanisms of action.

**Materials and methods:**

The expression of miR‐191 in HCC tissues was determined by quantitative real‐time PCR (qRT‐PCR). The role of miR‐191 in HCC cells was examined by using both in vitro and in vivo assays. Downstream targets of miR‐191 were determined by qRT‐PCR and Western blot analysis. Dual‐luciferase assays were performed to validate the interaction between miR‐191 and its targets.

**Results:**

The expression of miR‐191 was significantly higher in HCC patients and a higher miR‐191 expression predicted poorer prognosis. Analysis of The Cancer Genome Atlas data sets suggested that miR‐191 positively correlated with cell cycle progression. Gain and loss of function assays showed that miR‐191 promoted cell cycle progression and proliferation. Luciferase reporter assay showed that miR‐191 directly targeted the 3'‐untranslated region of KLF6 mRNA. Furthermore, circular RNA has_circ_0000204 could sponge with miR‐191, resulting in inactivation of miR‐191.

**Conclusions:**

Our study sheds light on the novel underlying mechanism of miR‐191 in HCC, which may accelerate the development of cancer therapy.

## 1 INTRODUCTION

Hepatocellular carcinoma (HCC) is common human malignancy worldwide, and its prevalence in Asia is higher when compared to that in other continents.^1^, ^2^, ^3^ HCC tumorigenesis is mainly accompanied by aberrant cell cycle progression, including cell cycle checkpoint inactivation, cyclins, and cyclins dependent kinases (CDKs) dysregulation.^1^, ^4^ Several studies suggested that cell cycle‐related genes were valuable therapeutic targets.^5^, ^6^, ^7^ However, the underlying mechanisms of cell cycle regulation involved in hepatocarcinogenesis have still not been elucidated. Therefore, it is crucial for us to identify the potential pathogenesis and molecular mechanism involved in cell cycle regulation in HCC.

MicroRNAs (miRNAs), as post‐transcriptional regulators of gene expression, exert their function by targeting 3'‐untranslated region (3'‐UTR) of protein‐coding genes, which are involved in regulating numerous biological processes.^8^ Accumulating evidence has suggested that altered miRNA levels are found in various types of human cancers and might play critical roles in tumorigenesis.^9^ miR‐191, a highly conserved miRNA, was found to be abnormally expressed in more than twenty different cancer types and has shown to be a major player in the regulation of some of these.^10^ In patients with HCC, miR‐191 was reported as a serum exosomal miRNA and a potential oncogenic target for HCC therapy.^11^, ^12^ In HCC cancer cells, miR‐191 was shown to be regulated mainly by DNA methylation and involved in the regulation of epithelial mesenchymal transition (EMT).^13^These studies suggested an important role of miR‐191 in HCC development. However, the molecular mechanisms by which miR‐191 exerted its effects in HCC tumorigenesis as well as its significance remain largely unknown.

Circular RNA is another type of non‐coding RNAs. In recent studies, circular RNAs have been reported to participate into tumorigenesis of various types of tumours, including HCC.^14^, ^15^ For example, circular RNA SMARCA5 inhibited the proliferation and migration of HCC cells by sponging with miR‐17‐3p and miR‐181b‐5p.^16^ Moreover, circular RNA circMTO1 was found to function as the sponge of miR‐9, thereby suppressing HCC progression.^17^ Circular RNA HIPK3 regulated cell proliferation and migration by sponging miR‐124 to increase Aquaporin‐3（AQP3）expression.^18^ These studies suggested that circular RNA functioned as a miRNA sponge in HCC cells.

Krüppel‐like factor (KLF) belongs to a family of zinc finger transcription factors that control essential cellular processes.^19^, ^20^ KLF6, a member of the KLF family, functions as an essential player in cell growth and cell cycle progression.^21^, ^22^ In cancer studies, KLF6 has been reported to mediate growth suppression by upregulation of p21, whereas downregulation of KLF6 activated c‐MYC transcription in prostate cancer.^23^, ^24^, ^25^ In HCC, KLF6 was reported to be downregulated in tumour tissues and resulted in cell cycle progression arrest and cell death.^26^, ^27^ Besides, Recent studies revealed that KLF6 suppressed the expression of cyclin D, resulting in G1 cell cycle arrest in hepatocellular carcinoma‐derived cells.^28^

## 2 MATERIALS AND METHODS

### 2.1 Cell culture and cell transfection

The Hep3B and HepG2 cells used in this study were obtained from Chinese Academy of Science. Cells were cultured in Dulbecco's modified Eagle's medium (DMEM), supplemented with 10% foetal bovine serum (FBS), 100 U/mL ampicillin and 100 g/mL streptomycin (Life Technologies), at 37℃, and 5% CO_2_. The HEK 293T cell line was used to produce lentivirus, and cells were transfected with pCDH‐pri‐miR‐191 or purogreen‐miZip‐191 (shRNA) or pZW‐hsa_circ‐0000204 or control plasmid and two package plasmids and then incubated for at least 10 hours at 37℃. For the selection of stable cell lines, overexpression or knock‐down lentivirus was transduced into liver cancer cells in the presence of polybrene (5μg/mL; Sigma), and cells were selected using medium containing 2 μg/mL puromycin for a total of two weeks. pcDNA‐KLF6 was transient transfection into liver cancer cells for expressing KLF6.

### 2.2 Clinical samples

All tissue samples used in this study were collected from the Affiliated Hospital of Nanjing University of Chinese Medicine. Written informed consent was obtained from all study participants. This study was approved by the Ethics Committee of Nanjing University of Chinese Medicine Authority.

### 2.3 Human HCC data set and bioinformatics analysis

In this study, HCC microarray data sets GSE10694 29 (CapitalBio Mammalian miRNA Array Services V1[1].0), GSE6857^30^ (OSU‐CCC MicroRNA Microarray Version 2.0), liver cancer TCGA(TCGA‐LIHC), (UNC_IlluminaHiSeq_RNASeqV2, level 3) and corresponding clinical data were downloaded from Gene Expression Omnibus (GEO) database (http://www.ncbi.nlm.nih.gov/geo/) and the open access tiers of the Cancer Genome Atlas (TCGA) data portal (https://gdc-portal.nci.nih.gov/). The KM plotter database (http://kmplot.com/analysis/) was used for the analysis of patients’ prognosis.^31^In order to gain insight into the biological mechanism involved in HCC progression through miR‐191, Pearson correlation tests were performed in tumour samples of the TCGA data sets.^32^ Moreover, pathway enrichment analysis was performed using Funrich software version 3.0 (http://funrich.org/index.html). HCC circular RNA microarray data sets GSE94508^33^ (Agilent‐069978 Arraystar Human CircRNA microarray V1) were downloaded from the GEO database. Differences in expression were analysed by GEO2R(https://www.ncbi.nlm.nih.gov/geo/geo2r). Circular‐miRNA prediction was performed by the 34 (https://circinteractome.nia.nih.gov/).

### 2.4 Edu assay

Cell proliferation was evaluated by Edu (5‐ethynyl‐20‐deoxyuridine) assay using a Cell‐Light EdU DNA Cell Proliferation Kit (RiboBio). In brief, Hep3B and HepG2 cells (1×10^3^) were seeded into wells of 8‐well plates. After incubation at 37℃ and 5% CO_2_ for 48 h, cells the proliferation rate was calculated according to the manufacturer's instructions. Representative images were taken using Eclipse Ni‐U fluorescence microscopy.

### 2.5 CCK‐8 assay

The CCK‐8 assay was performed in six repeated wells by using the Cell Counting Kit‐8 Assay kit (Doindo, Japan), following the manufacturer's guidelines. In brief, 1×10^3 ^cells were seeded in 96‐well plates, and the OD_450_ absorption value was measured using an automatic microplate reader (Synergy4; BioTek).

### 2.6 Colony formation assay

A total of 0.5×10^3 ^Hep3B and HepG2 cells were plated in wells of a 6‐well plate and cultured for about 2 weeks. Then, images were taken, and the number of colonies per well was counted by image J.

### 2.7 Cell cycle analysis

1×10^6 ^cells for flow cytometry analysis were seed into 100 cm^2 ^plate for 12 hours and then incubated in medium without FBS medium for 12 hours. Subsequently, cells were collected for flow cytometry, washed with phosphate‐buffered saline (PBS), and then fixed with cold 70% ethanol for at least 2 hours. Fixed cells were washed with PBS and incubated with RNAase A (0.1 mg/mL) and propidium iodide (50μg/mL) for 30 minutes at room temperature. Cell cycle analysis was performed using an Accuri C6 (BD Biosciences). For each analysis, 20,000 events were recorded. Data were analysed using ModFitLT V2.0 software (Becton Dickinson).

### 2.8 Western blot analysis

Western blot analyses were performed as previously described.^35^ Primary antibodies used were as follows: KLF6 (1:1000 dilution, sc‐20884; Santa Cruz Biotechnology), β‐actin (1:1000 dilution; Proteintech), CCND1(1:1000 dilution, 60186‐1‐Ig; Proteintech), CCND2(1:1000 dilution, 10934‐1‐AP; Proteintech), C‐MYC (1:1000 dilution, 10934‐1‐AP; Proteintech), CDK2 (1:1000 dilution, 10122‐1‐AP; Proteintech), CCNA2 (1:1000 dilution, 18202‐1‐AP; Proteintech)，CCNE (1:1000 dilution, 11554‐1‐AP; Proteintech).

### 2.9 RNA extraction and real‐time RT‐PCR

Total RNA was extracted using TRIzol reagent (Invitrogen). Targets were analysed by the SYBR Green qRT‐PCR assay according to the manufacturer's instructions (Applied Biosystems). The primers used are listed in Additional file Table S1. For miRNA detection, the reverse transcribed cDNA was synthesized using the All‐in‐One™ miRNA First‐Strand cDNA Synthesis Kit (GeneCopoeia). Next, miR‐191 expression was determined with the All‐in‐One™ miRNA qRT‐PCR Detection Kit (GeneCopoeia) and U6 snRNA was used as an endogenous control.

### 2.10 Dual‐luciferase reporter assay

The 3'‐UTR sequence of KLF6 containing wild type (WT), the mutant type (Mut), hsa_circ_0000204 containing wild type (WT) or mutant sequences of miR‐191 binding sites was inserted downstream of the luciferase reporter. The Dual‐Luciferase miRNA‐targets vector, pmirGLO, was obtained from Promega, Madison, USA. 3 × 10^3 ^cells were seeded into 96‐well plates and transfected with 50 nmol/L miR‐191 or negative control (NC) and 50 ng of the luciferase vector. Cells were harvested 48 hours after the transfection. The relative luciferase activity was measured by the Dual‐Glo luciferase assay kit (Promega).

### 2.11 RNA pull‐down assay

The RNA pull‐down lysates from HepG2 and SMMC7721 cells were incubated with biotin (Bio)‐labelled oligonucleotide miR‐191 (Bio‐5′‐GCTGCGCTTGGATTTCGTCCCC‐3′) or scramble probes (RiboBio, Guangzhou, China) for 2 hours at room temperature. Probes associated RNAs complexes were captured with Streptavidin‐coupled Dynabeads (60210, thermo). The complexes were incubated with Pulldown wash buffer (Millipore) containing proteinase K for 1 hours at 25°C. has_circ_0000204 and GAPDH (negative control) in the pull‐down were determined using qRT‐PCR analysis. The calculation formula is as follows：Bio‐miR‐191 pull‐down for hsa_circ_0000204/Scramble control pull‐down for hsa_circ_0000204 = X. Bio‐miR‐191 input/Scramble control input = Y. Enrichment Fold changes = X/Y.

### 2.12 Animal studies

All animal studies were performed in accordance with protocols approved by the Animal Experimentation Ethics Committee of Nanjing University of Chinese Medical Center. For tumour growth analysis in a xenograft model, 5‐6 weeks old immunodeficient mice were used. A total of 5 × 10^5^ cells were subcutaneously injected into the right collar of the mice (n = 4 per group). Tumour growth was analysed by measuring tumour length (L) and width (W) and by calculating the volume (V) using the formula, V = L × W^2^/2. Immunohistochemical analysis for targets was performed as previously described.^35^

### 2.13 Statistical analysis

Statistical analyses were conducted using SPSS16.0 or GraphPad Prism 5.0 software. Comparisons between groups were analysed by the t test, and multiple group comparisons were analysed using one‐way ANOVA *P* < 0.05 was considered statistically significant. Data were presented as the mean ± standard deviation (SD).

## 3 RESULTS

### 3.1 miR‐191 is upregulated and positively correlated with cell cycle progression in hepatocellular carcinoma tissues

**Figure 1:**
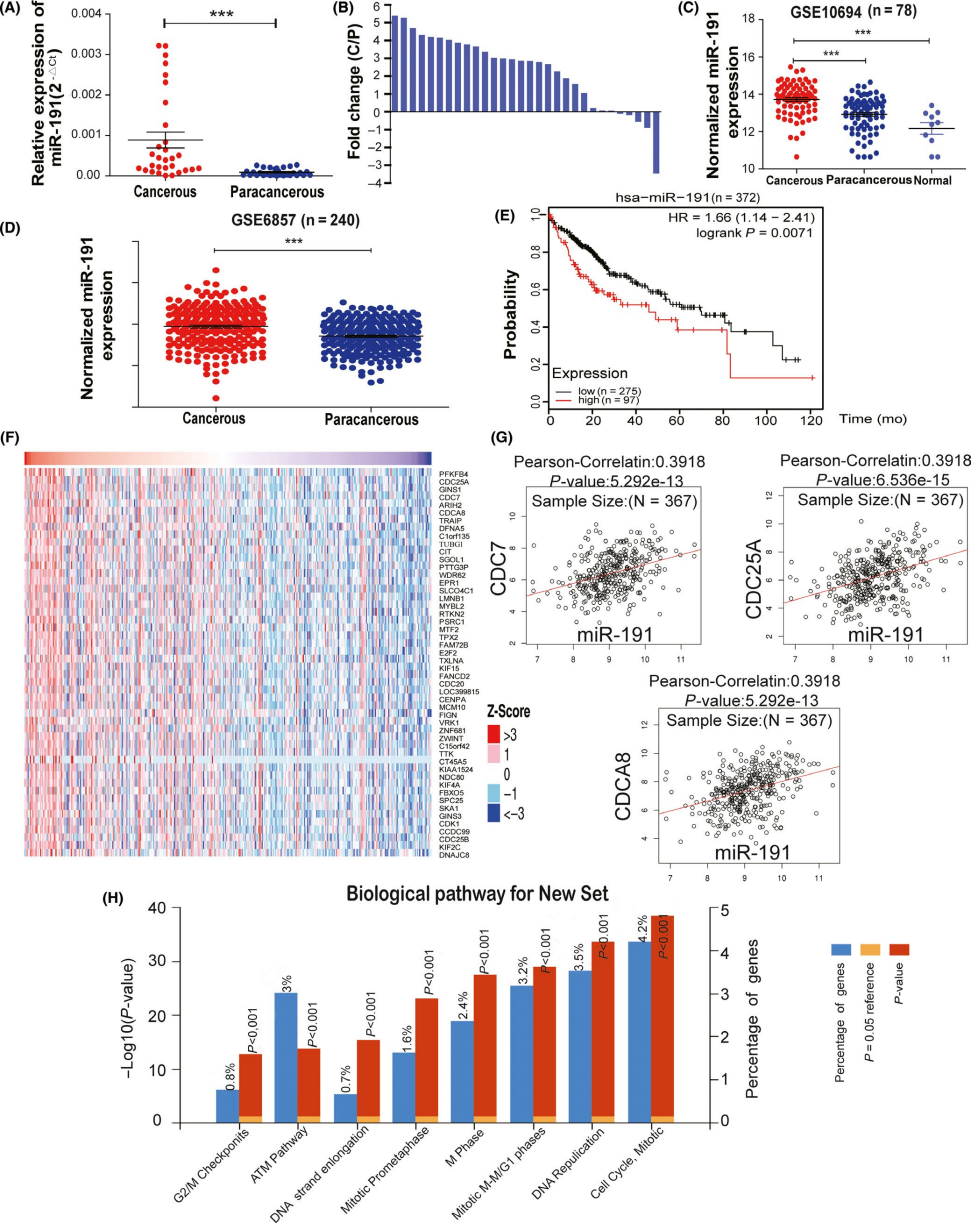
miR‐191 is upregulated and positively correlated with cell cycle progression in hepatocellular carcinoma tissues. A, Expression levels of miR‐191 in 30 pairs of HCC tissues and adjacent non‐tumour tissues. The data were analysed by a minus delta Ct method. B, Bars represent the relative miR‐191 expression comparing expression in HCC tissues C, versus adjacent non‐tumour tissues (P) using alogarithmic scale. C, D, Expression levels of miR‐191 in 78 paired, 240 paired HCC tissues and 10 normal tissues, respectively, obtained from the GEO database (GSE 10694 and GSE6857). E, The prognostic significance of miR‐191 for 372 HCC patients assessed by Kaplan‐Meier analysis (logrank *P* = 0.0071). F, Expression level of genes positively correlated with miR‐191 in HCC tissues obtained from TCGA LIHC data sets. G, The positive correlation between the expression of miR‐191 and cell cycle related genes (such as CDC7, CDC25A and CDCA8) in liver cancer TCGA data sets. H, The most five significant biological pathways were enriched by miR‐191 positive correlation genes (***P < 0.001)

To determine the role of miR‐191 in HCC tumorigenesis, we first examined miR‐191 expression levels in HCC tissues. The data showed that miR‐191 was highly expressed in HCC specimens when compared to adjacent non‐cancerous tissues (n = 30) (Figure 1A,B and Table 1). In addition, based on the analysis of GEO data sets, GSE10694^29^ and GSE6857,^30^ we found that miR‐191 was frequently upregulated in HCC tissues (Figure 1C,D). Next, we analysed miR‐191 expression levels with patients’ prognosis using KM plotter database (http://kmplot.com/analysis/) and demonstrated that patients with a higher miR‐191 expression in their tumour tissues had significantly shorter survival when compared to patients with lower miR‐191 expression (Figure 1E). Interestingly, we discovered that miR‐191 strongly and positively correlated with several important cell cycle regulators, including CDC25A, CDC7 and CDCA8 in the TCGA liver cancer database (Figure 1F,G). Enrichment of positively correlated genes revealed that the cell cycle progression was the most significant pathway (Figure 1H), which suggested that miR‐191 could be involved in regulation of cell cycle progression. Taken together, these results indicated that miR‐191 was upregulated in HCC tissue, correlated with cell cycle progression and that a higher expression of miR‐191 predicted poor prognosis.

**Table 1:** Correlation between miR‐191 expression and HCC clinicopathological parameters

Characteristic		miR‐191		*P* value
		Low = 15	High = 15	
Sex				
Male	26	12	14	0.716
Female	4	2	2	
Age				
<50	7	2	5	0.747
≥50	23	12	11	
Cirrhosis				
Absent	9	7	2	0.294
Present	21	7	14	
Node metastasis				
Absent	25	12	13	0.959
Present	5	2	3	
Histological grade				
I, II	17	9	8	0.298
III, IV	13	5	8	
Tumour size (cm)				
<5	16	9	7	0.708
≥5	14	5	9	
APF				
<50	20	10	10	0.070
≥50	10	4	6	

Abbreviation: AFP*,* alpha‐fetoprotein.

### 3.2 Knock‐down of miR‐191 suppresses cell cycle progression and cell proliferation in vitro

**Figure 2:**
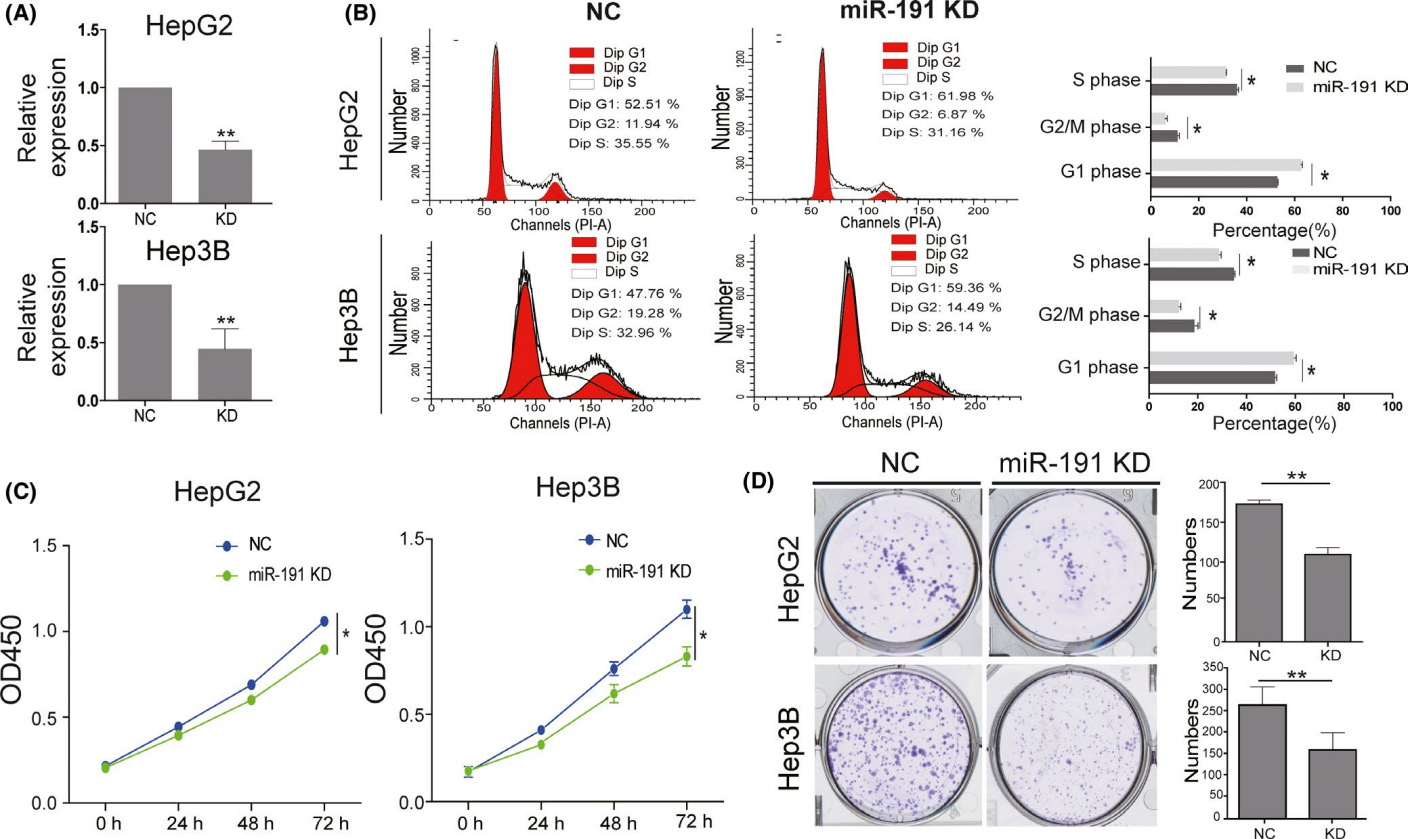
Knock‐down of miR‐191 suppresses cell cycle progression and cell proliferation in vitro. A, qRT‐PCR data showing that miR‐191 was significantly decreased in HepG2 and Hep3B cells with miR‐191 knock‐down plasmids transfected. B, Cell cycle analysis of HepG2 and Hep3B cells aftermiR‐191 knock‐down. C, The cell viability of HepG2 and Hep3B cells in which miR‐191 was knocked down was determined by CCK‐8 assays. D, Representative images of colonies of HepG2 and Hep3B control cells and miR‐191 depleted cells (*P < 0.05, **P < 0.01)

**Figure 3:**
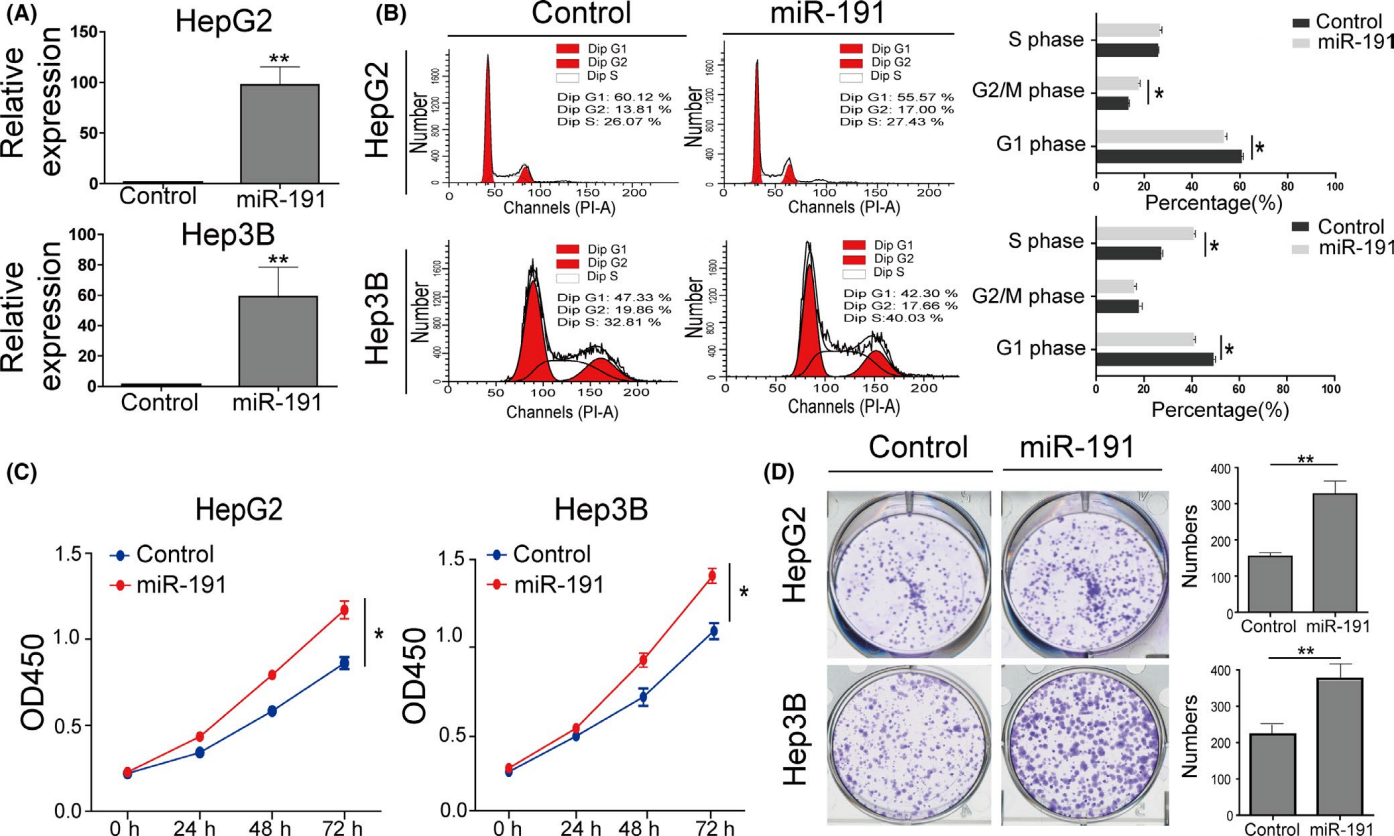
Enhancing miR‐191 levels promotes cell cycle progression and cell proliferation. A, qRT‐PCR data showing that miR‐191 was significantly upregulated in HepG2 and Hep3B cells with miR‐191 overexpression plasmids transfected. B, Cell cycle analysis of HepG2 and Hep3B cells in which miR‐191 is overexpressed. C, The cell viability of HepG2 and Hep3B cells with miR‐191 overexpression was determined by CCK‐8 assay. D, Colony formation assays were performed using HepG2 and Hep3B cells with miR‐191 overexpression

To further investigate miR‐191 function on HCC, we knocked down miR‐191 in Hep3B and HepG2 cells using shRNA‐miR‐191 plasmids. The results showed that miR‐191 was significantly decreased in Hep3B and HepG2 cells when transfected with miR‐191 shRNA plasmids (Figure 2A). According to the analysis of the liver cancer TCGA database above, we firstly performed flow cytometry assays to determine cell cycle progression. Analysis of the results showed that a reduction in miR‐191 significantly increased the proportion of cells in the G1 phase and decreased cells in the S and G2/M phase (Figure 2B). In addition, CCK‐8 assay results showed that a decrease in miR‐191 reduced cell viability (Figure 2C). Colony formation assay results also suggested that a reduction in miR‐191 impaired the ability of monoplasts to form colonies (Figure 2D). Next, we investigated the effects of enhancing miR‐191 expression. The results showed that enhancing miR‐191 expression promoted cell cycle progression and cell proliferation (Figure 3A‐D). The Western blot assay for CCNA2, CCNE1 and CDK2 also confirmed that upregulated miR‐191 could exert its effort on cell cycle progression (Figure S1A). Combined, our results demonstrated that miR‐191 had a positive effect on G1 phase to S/G2M phase transition and proliferation in vitro.

### 3.3 miR‐191 affects cell proliferation in vivo

**Figure 4:**
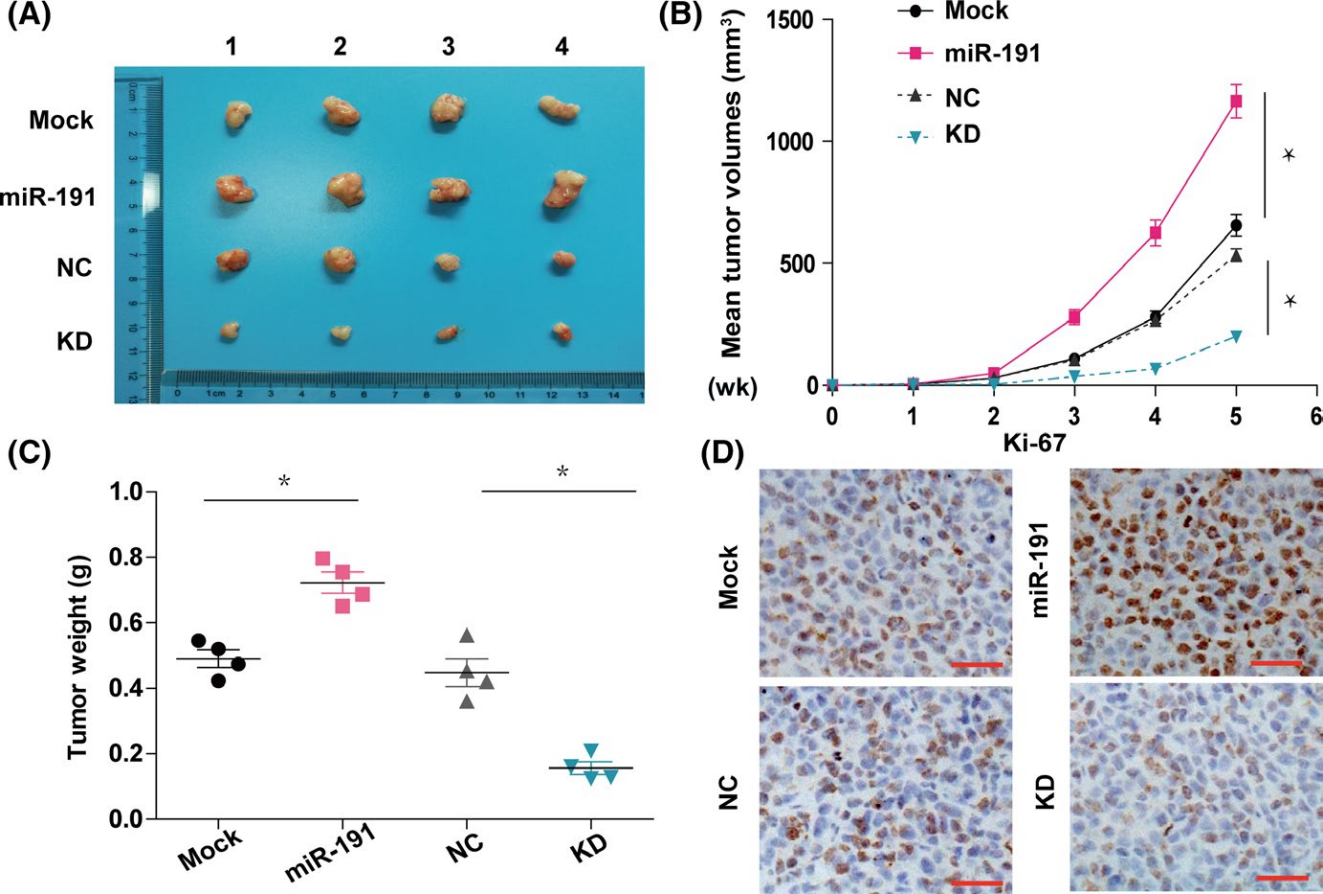
miR‐191 affects cell proliferation in vivo. A,B, Representative images of harvested tumours and quantitation of tumour weights at 35 days after injection with miR‐mock (Mock) HepG2 cells, cells with miR‐191 overexpression, negative control (NC) or miR‐191 knock‐down cells (KD); n = 4 mice per group. C, After subcutaneous injection in nude mice, tumour volume was calculated from day 1 to day 35. D, Immunohistochemical staining of Ki‐67 positive tumour cells in xenograft tumours from the four indicated groups. Representative images are shown, scale bar: 20 μm

To explore the effects of miR‐191 in vivo, we established a mouse xenograft model to investigate whether miR‐191 could promote tumour growth. HepG2 cells overexpressing or knocked down for miR‐191 were subcutaneously injected into nude mice. We demonstrated that after 35 days, the tumour size was decreased in the miR‐191 knock‐down group when compared with the control group (Figure 4A,B). Similar results were obtained for tumour weight (Figure 4C). Moreover, tumour sections from the miR‐191 knock‐down group exhibited weaker Ki67 staining when compared to those from the control group, suggesting that knock‐down of miR‐191 inhibited tumour growth (Figure 4D). In addition, opposite effects were obtained from the miR‐191 overexpression group. Thus, these results showed that miR‐191 promoted tumour growth in vivo.

### 3.4 KLF6 is the downstream target that is directly regulated by miR‐191 in HCC

**Figure 5:**
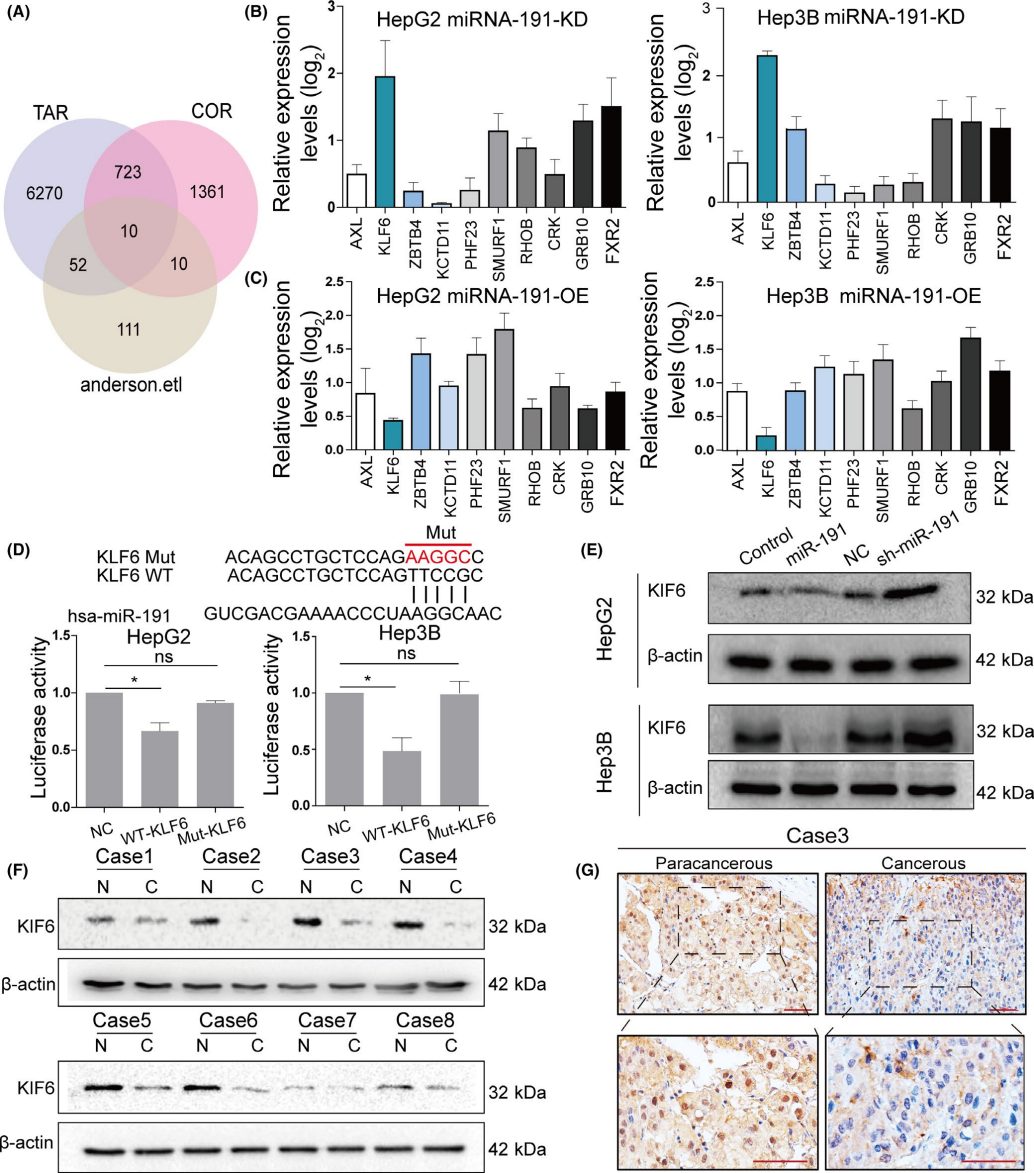
Identification of the targets directly regulated by miR‐191 in hepatocellular carcinoma. A, Venn diagram of overlapped genes in microarray data (upregulated, fold change ≥ 2.0), TCGA data (negatively related, *r* < −0.1), target prediction analysis (TarPmiR algorithm, *P* < 0.05). B,C, mRNA expression levels of the indicated genes in HepG2 and Hep3B cells in which miR‐191 was knocked down (KD) or overexpressed (OE). D, Predicted binding sites of 3'‐UTR of KLF6 to miR‐191, and the relative luciferase activities in different groups (* *P* < 0.05). E, Protein expression levels of the indicated genes in HepG2 and Hep3B cells in which miR‐191 was overexpressed or knocked down. F ,Western blot analysis of KLF6 in eight pairs of HCC tissues (N: indicated non‐tumour tissues; C: indicated HCC tissue). G, KLF6 protein levels in HCC tissues and adjacent non‐tumour tissues by immunohistochemical analysis, scale bar: 20 μm

Having elucidated the functional phenotype of miR‐191 in HCC, we next identified its targeting genes to gain further insights into the molecular mechanism of miR‐191 on HCC cells. To our knowledge, miRNAs usually function as a gene silencer, suppressing expression of its targets.^36^ Here, we primarily found negatively correlated genes of miR‐191 in the liver cancer TCGA database. Our analysis showed that a total of 2202 transcripts negatively correlated with miR‐191(*r* < −0.1). We searched for miR‐191 targeted genes using miWalk 3.0, an integrated database predicted gene‐miRNA interaction and the results showed that there were 7055 predicted targets. Moreover, we also found a data set that demonstrated that 178 transcripts were upregulated after knock‐down of miR‐191 in HepG2.^11^ These three data sets were overlapped to identify the genes that met the following criteria: (a) potential targets of miR‐191, (b) increased expression by miR‐191 knock‐down and (c) negatively correlated with miR‐191 in liver cancer tissues. Ultimately, our analysis showed that only 10 transcripts met the criteria (Figure 5A). To confirm the targets directly regulated by miR‐191, we performed qRT‐PCR experiments to determine the mRNA expression of these candidate transcripts in cell lines with miR‐191 overexpressing or miR‐191 deficiency. The results showed that only KLF6 was increased in cells with miR‐191 deficiency and was decreased in miR‐191 overexpressing cells (Fold changes > 2, *P* values < 0.05) (Figure 5B,C). Dual‐luciferase reporter assays further confirmed the direct interaction of miR‐191 and KLF6 mRNA (Figure 5D). In addition, Western blot analyses were performed to confirm the relationship between miR‐191 and KLF6 (Figure 5E). Next, we determined the correlation between miR‐191 levels and KLF6 protein levels in 8 paired HCC tissues. The results showed that miR‐191 negatively correlated with KLF6 protein expression in HCC tissues (Figure 5F and Figure S1B,C). Immunohistochemical analyses further confirmed that KLF6 protein negatively correlated with miR‐191 expression (Figure 5F). Together, these findings suggested that KLF6 was a direct target of miR‐191.

### 3.5 KLF6 mediates regulation of miR‐191 on cell cycle and cell proliferation

**Figure 6:**
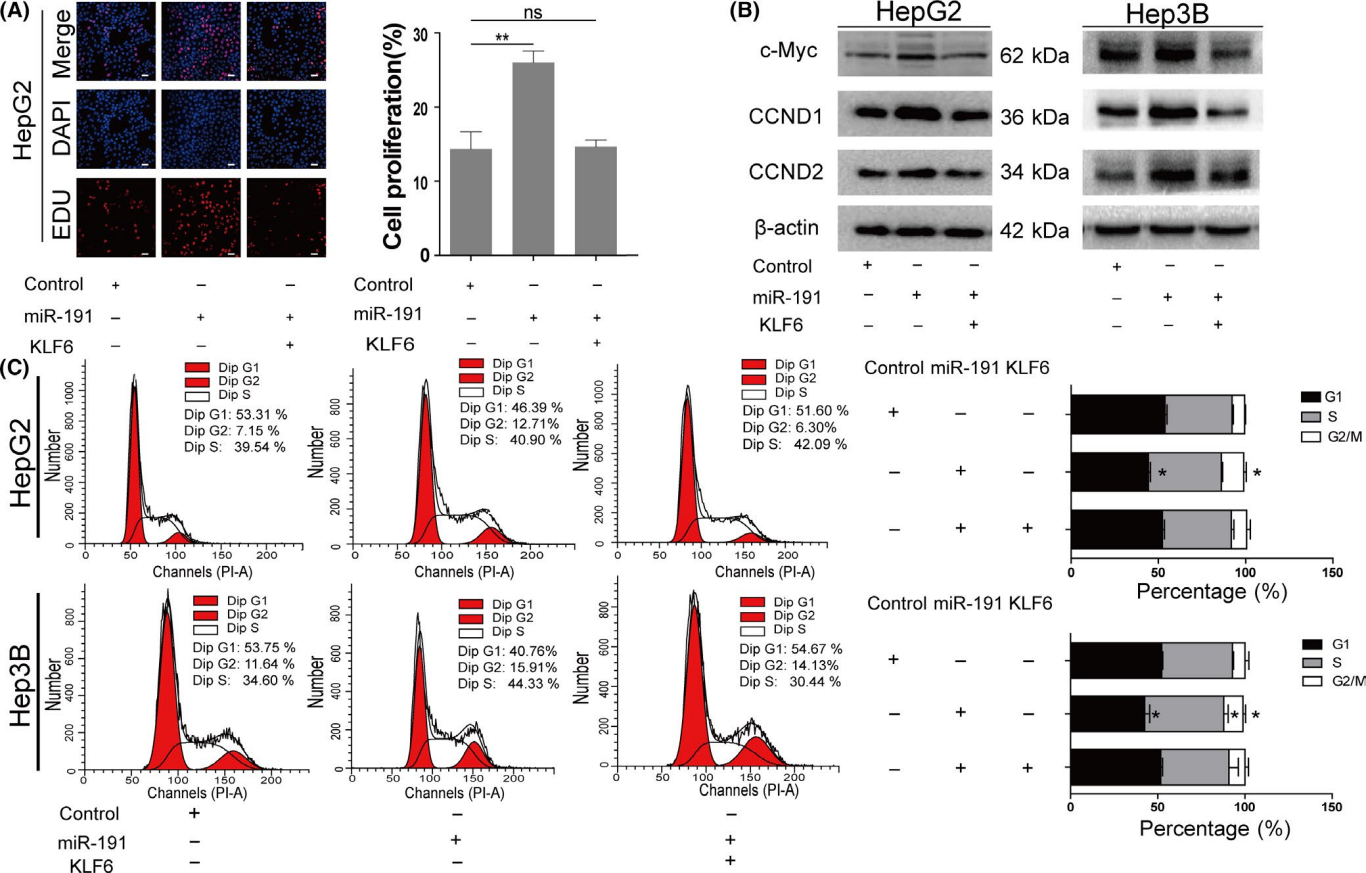
KLF6 mediates miR‐191 regulation of cell cycle phase and cell proliferation. A, Edu assays detected HepG2 and Hep3B cells with the group of miR‐191 deficiency and KLF6 overexpression. B, Cell cycle analysis of HepG2 and Hep3B cells with miR‐191 deficiency and KLF6 overexpression. C, mRNA and protein levels of the indicated genes after KLF6 overexpression in HepG2 and Hep3B cells with miR‐191 deficiency

Based on our results, we hypothesized that KLF6 directly mediated miR‐191‐regulated cancer cell proliferation. To further elaborate on this critical issue, we forced KLF6 expression in HepG2 cells overexpressing miR‐191. The ectopic KLF6 expression in the miR‐191‐transduced cells attenuated the proliferative effects of miR‐191 on HepG2 proliferation (Figure 6A). Cell cycle‐related protein levels, which are regulated by KLF6 were also changed as we expected, such as c‐Myc and CCND1/CCND2 (Figure 6B). Similarly, the increase in KLF6 blocked the promotion of cell cycle progression accelerated by miR‐191 (Figure 6C). Taken together, these results indicated that KLF6 mediated miR‐191 promotion of HCC cell cycle progression and proliferation.

### 3.6 miR‐191 is sponged by circular RNA has_circ_0000204

**Figure 7:**
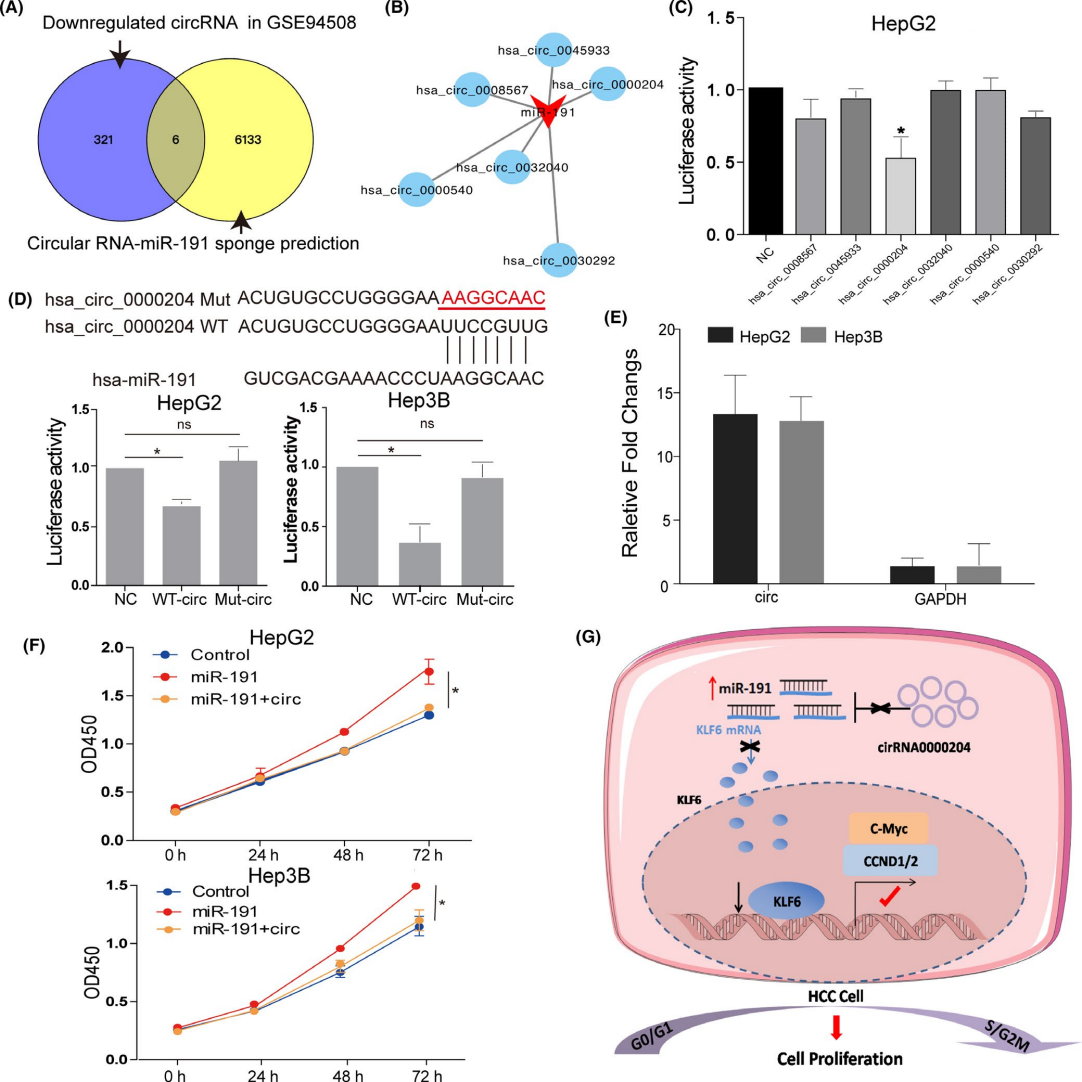
miR‐191 is sponged by circular RNA has_circ_0000204. A, Venn diagram of overlapped circular RNAs in GEO data sets, GSE94508 (downregulated, fold change ≥ 2.0), circular RNAs‐ miRNA binding analysis (circinteractome, *P* < 0.05). B, The six circular RNAs had the potential binding site with miR‐191 in the circular RNA profile of five paired HCC patients. C, The relative luciferase activities in different groups (*P < 0.05). D, Predicted binding sites of miR‐191 and has_circ_0000204, and luciferase activities in the group of miR‐191 and hsa_circ_0000204. E, RNA pull‐down assay for the interaction between miR‐191 and has_circ_0000204 in Hep3B and HepG2. The calculation formula is as follows: Bio‐miR‐191 pull‐down for hsa_circ_0000204/Scramble control pull‐down for hsa_circ_0000204 = X. Bio‐miR‐191 input/Scramble control input = Y. Fold binding = X/Y. F, The cell viability of HepG2 and Hep3B cells control group, miR‐191 overexpression group, or miR‐191 overexpression combined with forced has_circ_0000204 expression group was determined by CCK‐8 assay (*P < 0.05). G, Schematic illustration of the mechanisms underlying has_circ_0000204/miR‐191/KLF6 network

Recent studies suggested that miRNA could be sponged by circular RNA, resulting in miRNA inactivation.^37^ According to GEO data sets GSE94508,^33^ a circular RNA profile of five paired HCC patients, there were six circular RNAs had the potential binding site with miR‐191 and downregulated in HCC tissues (Figure 7A,B). Next, we performed luciferase assays to identify which circular RNA could bind with miR‐191. The results showed that the group of has_circ_0000204 and miR‐191 could decrease the most of luciferase activities compared with others (Figure 7C), while the group of has_circ_0000204mut (mutated bind sequence) and miR‐191 could not influence luciferase activities significantly, suggesting that has_circ_0000204 could act as miR‐191 sponge (Figure 7D). Then，we used biotin labelled miR‐191 pull‐down assay to identify whether miR‐191 could directly bind has_circ_0000204. qRT‐PCR analysis of the expression levels of has_circ_0000204 or GAPDH（negative control）in the HepG2 and Hep3B lysates after biotin‐miR‐191 pull‐down assay. Overall, we found a specific enrichment (13.8 times fold changes for HepG2 and 12.7 times fold changes for Hep3B) of has_circ_0000204 (Figure 7E). Using functional assays, we demonstrated that has_circ_0000204 weaken the proliferous phenotype of high expression of miR‐191(Figure 7F). These results illustrated that miR‐191 could be inactivated by sponging with has_circ_0000204 (Figure 7G).

## 4 DISCUSSION

Dysregulation of cell cycle progression is an essential step in tumorigenesis and the development of various human cancers.^4^ Several studies revealed that miR‐191 controlled major proliferation pathways by regulating critical cell cycle regulators in other cancers.^10^ For example, the inhibition of miR‐191 induced gastric cancer cells to be decreased in the S phase.^38^ miR‐191 also promoted colorectal cancer cells cycle progression by targeting CCAAT enhancer binding proteins (C/EBPβ) expression, thereby increasing the proportion of cells in the S phase as well as increasing the tumorigenesis capability of colorectal cancer.^39^ However, Yendamuriet al. reported that miR‐191 could not alter cell proliferation and cell cycle progression in BEAS‐2B and A549 lung cancer cell lines.^40^ These findings suggested an important phenomenon that the cell cycle progressions of cancer cells were controlled by miR‐191 although arguments existed. In our study, we found that the inhibition of miR‐191 suppressed the G1‐S/G2M transition mediated by the transcription factor KLF6. In previous HCC studies, KLF6 has been reported as a tumour suppressor.^21^ Reduced KLF6 expression was common in both hepatitis B virus (HBV)—and hepatitis C virus (HCV) ‐related HCCs and occurs at critical stages during cancer progression. The effects of KLF6 can be attributed to the regulation of genes controlling hepatocyte growth and differentiation.^23^ In other cancers, it was demonstrated that upregulation of KLF6 inhibited cell cycle progression, which was consist of our findings.

In HCC, miR‐191 was highly expressed in HCC tissues and a higher expression of miR‐191 predicted poorer survival.^11^ In our studies, we expanded the number of samples to identify these views by using tissues we collected as well as by the GEO database data sets we analysed. As expected, our results were consisted with the data presented previously. However, a few studies were performed revealing the regulatory mechanism of miR‐191 upregulation. Yinghuaet al. proposed that miR‐191 was upregulated due to promoter hypomethylation, and hypomethylation of miR‐191 promoted miR‐191 expression, thus, induced HCC cell EMT.^13^ Chen at al. suggested that miR‐191 was regulated by HIF‐2, resulting in EMT.^41^ In our study, we were likely to propose that miR‐191 was inactivated by circular RNA hsa_circ_0000204 at the posttranscriptional level. Our results supplemented the regulator mechanism of miR‐191 upregulation, which illustrated the onco‐miRNA role in HCC.

Circular RNAs have been reported to play an important role in tumorigenesis and tumour development.^42^ Previous studies revealed that circular RNA mainly played a role as a sponge for miRNA inactivation.^37^ This hypothesis was in part derived from the circular RNA structure. Due to the circular structure, and by linking the 5' end and 3'end, we avoided degradation by RNase. Therefore, circular RNA was more stable when compared with liner transcripts, possessing the ability of a sponge.^37^ In our study, we selected circular RNA has_circ_0000204 because of the binding site for miR‐191 and the negative expression pattern between them. Towards this, we speculated that has_circ_0000204 could sponge with miR‐191. However, we understand that our methods could not explore all circular RNAs to influence miR‐191 to alter cellular function in HCC. In addition, we also believed that the function of has_circ_0000204 was not limited to regulation of cell cycle and cell proliferation because more than one miRNA binding site was present.

## 5 CONCLUSIONS

The inspiration of this study was that miR‐191 expression markedly and positively correlated with cell cycle regulators in HCC tissues. Enhancing expression of miR‐191 promoted the cell cycle progression and proliferation of HCC cells, whereas inhibition of miR‐191 expression had the opposite effect. Moreover, we demonstrated that miR‐191 upregulation in HCC cells suppressed both KLF6 mRNA and protein levels, thereby inhibiting expression of its targets and promoting G1‐S/G2M phase transition. In addition, has_circ_0000204 regulated miR‐191 by acting as a miR‐191 sponge. Our results indicated that miR‐191 partially promoted HCC tumorigenesis and development by regulating cell cycle progression.

## CONFLICT OF INTEREST

The authors declare no conflicts of interest.

## AUTHOR CONTRIBUTIONS

FT, LFW and XJZ conceived and coordinated the study. FT, MHS, MW and XYW carried out the experiments. CTY analysed the data. FT and LFW wrote the manuscript. All authors have read and approved the final manuscript. FT, CTY and MHS were co‐first authors.
