# Supplementary figures and images for: MicroRNA‐191 promotes hepatocellular carcinoma cell proliferation by has_circ_0000204/miR‐191/KLF6 axis
Source: Cell Prolif. 2019 Jul 23;52(5):e12635. doi: 10.1111/cpr.12635 (PMC6797514; doi:10.1111/cpr.12635)

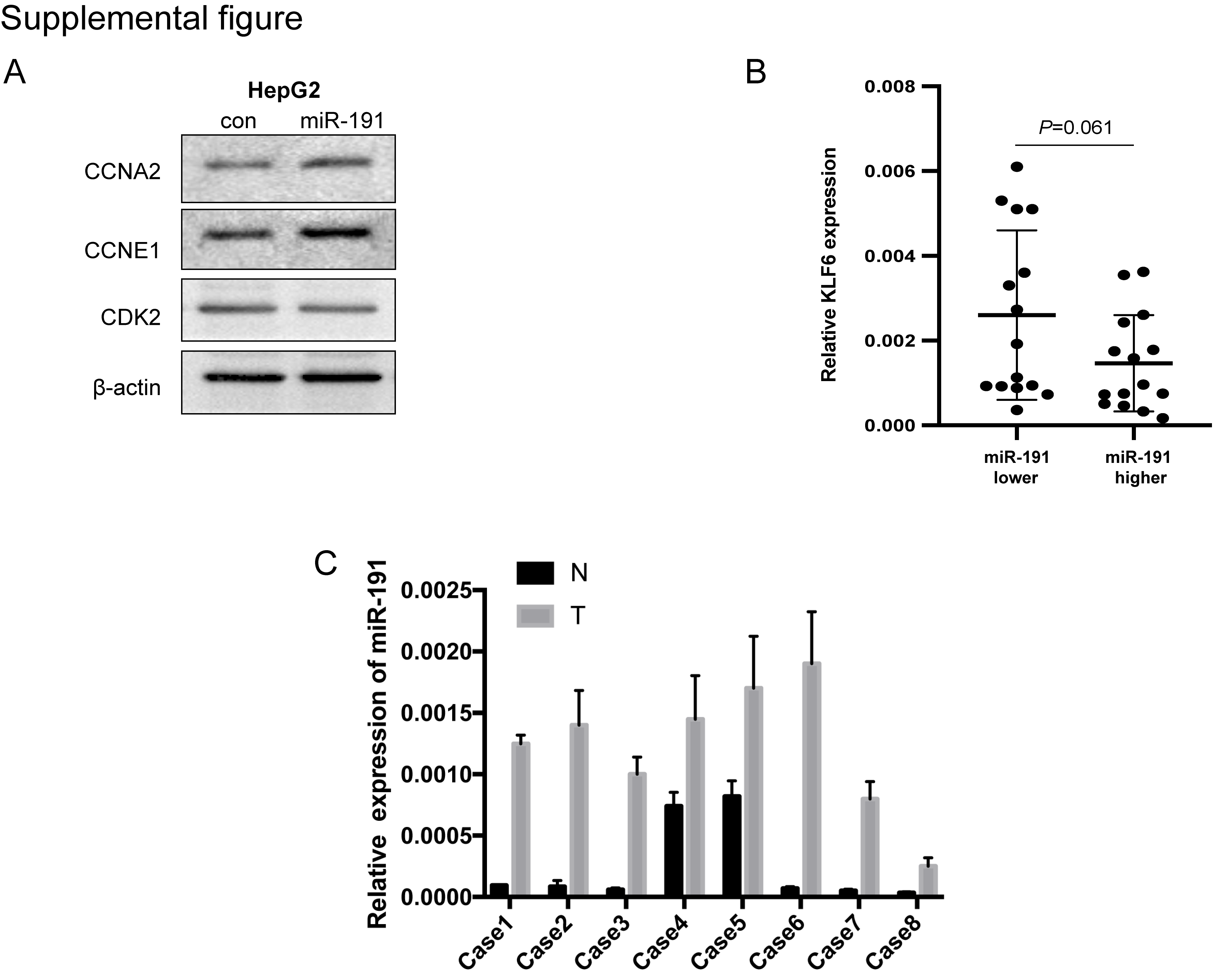

Supplement: Supplementary file 1 [file CPR-52-e12635-s001.tif]
