# Supplementary material for: MicroRNA‐191 promotes hepatocellular carcinoma cell proliferation by has_circ_0000204/miR‐191/KLF6 axis
Source: Cell Prolif. 2019 Jul 23;52(5):e12635. doi: 10.1111/cpr.12635 (PMC6797514; doi:10.1111/cpr.12635)
Supplement: Supplementary file 2 [file CPR-52-e12635-s002.docx]

| **Supplemental_Table_1** | **Primer sequences used in this study** |
| --- | --- |
| Primers | Sequence |
| Axl-F | CAGTGCCAAATCCGGGGAG |
| Axl-R | AGCATCCTGGAGCCAGAGTA |
| ZBTB4-F | TCCCTTTTGCACTGAGGCTT |
| ZBTB4-R | AGAAGGGACTTGAAGCAGCC |
| KLF6-F | GGCCAAGTTTACCTCCGACC |
| KLF6-R | TAAGGCTTTTCTCCTTCCCTGG |
| KCTD11-F | GGGAGGAGAGAGAATGGGGT |
| KCTD11-R | TCCAGATGAGGGATGCTCCA |
| PHF23-F | GGACACTTCTGTGGAGCGAA |
| PHF23-R | CGTTTCTCTGGTGGCTGAGT |
| SMURF1-F | GCTTTGCAAGGCGCGG |
| SMURF1-R | TGGGAGCCACCAACAAAAGT |
| RHOB-F | CAGTAAGGACGAGTTCCCCG |
| RHOB-R | GTCCACCGAGAAGCACATGA |
| CRK-F | TTGAGAATCCGGGACAAGCC |
| CRK-R | GGCCCATTCTGGAGGTTAGG |
| GRB10-F | TCCAAGCGGTGCATTCTTGA |
| GRB10-R | GCTAACGGGTCACCAACAGA |
| FXR2-F | GCTCTATCAGTGGAGACCGC |
| FXR2-R | GGACAAGAGGGAGGGGGTAT |
| mir-191-F | CAACGGAATCCCAAAAGCAGCTG |
| mir-191-R | provided by kit |
| sh-miR-191-F | gatcccaacggaatcccaaaagcagctgcttcctgtcagacagctgcttttgggattccgttgtttttg |
| sh-miR-191-R | aattcaaaaacaacggaatcccaaaagcagctgtctgacaggaagcagctgcttttgggattccgttgg |
| has-mir-0000204-F | ACTAACAGCTCGGAGCAGGA |
| has-mir-0000204-R | CCAACATGCCTCCATCATCT |
| pri-miR-191-EcoR1 | atgaattcCTCTAGACTCCGTTTCACAACC |
| pri-miR-191-BamH1 | atggatccTCCAGAGATGGCCACCAGC |
| has-mir-0000204-Xhol1 | atctcgagAACCATCCCCTGCTCTTGGG |
| has-mir-0000204-Aflll | atcttaagTTGTCCTTCTAACATGTCTCTTG |
| KLF6-EcoR1 | atgaattcATGGACGTGCTCCCCATGTG |
| KLF6-BamH1 | atggatccTTGTCCTTCTAACATGTCTCTTGGC |
| luc-KLF6-F | AAAC TA GCGGCCGC TAGT ACAGCCTGCTCCAGTTCCGC T |
| luc-KLF6-R | CTAGAGCGGAACTGGAGCAGGCTGTACTAGCGGCCGCTAGTTT |
| luc-0000204-F | AAAC TA GCGGCCGC TAGT ACTGTGCCTGGGGAATTCCGTTG T |
| luc-0000204-R | CTAGACAACGGAATTCCCCAGGCACAGTACTAGCGGCCGCTAGTTT |
| luc-0008567-F | AAAC TA GCGGCCGC TAGTTGCTGCAGAAGCGTATCCGTTAC T |
| luc-0008567-R | CTAGAGTAACGGATACGCTTCTGCAGCAACTAGCGGCCGCTAGTTT |
| luc-0045933-F | AAAC TA GCGGCCGC TAGTAACCAAACTCAGCCCTCCGTTAA T |
| luc-0045933-R | CTAGATTAACGGAGGGCTGAGTTTGGTTACTAGCGGCCGCTAGTTT |
| luc-0032040-F | AAAC TA GCGGCCGC TAGTTGCCAGGAAATTCTCTCCGTTAC T |
| luc-0032040-R | CTAGAGTAACGGAGAGAATTTCCTGGCAACTAGCGGCCGCTAGTTT |
| luc-0000540-F | AAAC TA GCGGCCGC TAGTTTTTTTGTAAATAAATCCGTTAT T |
| luc-0000540-R | CTAGAATAACGGATTTATTTACAAAAAAACTAGCGGCCGCTAGTTT |
| luc-0030292-F | AAAC TA GCGGCCGC TAGTGTGAGCTTTCCGTTG T |
| luc-0030292-R | CTAGACAACGGAAAGCTCACACTAGCGGCCGCTAGTTT |
